# A Quality Improvement Intervention to Inform Scale-Up of Integrated HIV-TB Services: Lessons Learned From KwaZulu-Natal, South Africa

**DOI:** 10.9745/GHSP-D-21-00157

**Published:** 2021-09-30

**Authors:** Santhanalakshmi Gengiah, Kogieleum Naidoo, Regina Mlobeli, Maureen F. Tshabalala, Andrew J. Nunn, Nesri Padayatchi, Nonhlanhla Yende-Zuma, Myra Taylor, Pierre M. Barker, Marian Loveday

**Affiliations:** a Centre for the AIDS Programme of Research in South Africa (CAPRISA), Durban, South Africa.; b Medical Research Council-CAPRISA HIV-TB Pathogenesis and Treatment Research Unit, Doris Duke Medical Research Institute, University of KwaZulu-Natal, Durban, South Africa.; c Institute for Healthcare Improvement, Cambridge, MA, USA.; d Medical Research Council, Clinical Trials Unit at University College London, London, United Kingdom.; e School of Nursing and Public Health, University of KwaZulu-Natal, Durban, South Africa.; f Department of Maternal and Child Health, Gillings School of Global Public Health, University of North Carolina, Chapel Hill, NC, USA.; g HIV Prevention Research Unit, South African Medical Research Council, South Africa.

## Abstract

**Introduction:**

In South Africa, mortality rates among HIV-TB coinfected patients are among the highest in the world. The key to reducing mortality is integrating HIV-TB services, however, a generalizable implementation method and package of tested change ideas to guide the scale-up of integrated HIV-TB services are unavailable. We describe the implementation of a quality improvement (QI) intervention, health systems’ weaknesses, change ideas, and lessons learned in improving integrated HIV-TB services.

**Methods:**

Between December 1, 2016, and December 31, 2018, 8 nurse supervisors overseeing 20 primary health care (PHC) clinics formed a learning collaborative to improve a set of HIV-TB process indicators. HIV-TB process indicators comprised: HIV testing services (HTS), TB screening among PHC clinic attendees, isoniazid preventive therapy (IPT) for eligible HIV patients, antiretroviral therapy (ART) for HIV-TB coinfected patients, and viral load (VL) testing at month 12. Routine HIV-TB process data were collected and analyzed.

**Results:**

Key change interventions, generated by health care workers, included: patient-flow redesign, daily data quality checks; prior identification of patients eligible for IPT and VL testing. Between baseline and post-QI intervention, IPT initiation rates increased from 15.9% to 76.4% (*P*=.019), HTS increased from 84.8% to 94.5% (*P*=.110), TB screening increased from 76.2% to 85.2% (*P*=.040), and VL testing increased from 61.4% to 74.0% (*P*=.045). ART initiation decreased from 95.8% to 94.1% (*P*=.481).

**Discussion:**

Although integrating HIV-TB services is standard guidance, existing process gaps to achieve integration can be closed using QI methods. QI interventions can rapidly improve the performance of processes, particularly if baseline performance is low. Improving data quality enhances the success of QI initiatives.

## Abstract (precis)

Despite being standard of care, gaps in HIV-TB service delivery are present. Quality Improvement methods are effective in uncovering health systems weaknesses that impede efficient delivery of integrated HIV-TB services.

## Teaser

**Key Findings:**

- The quality improvement (QI) intervention was able to guide clinic staff in developing simple but effective change interventions, using resources already available, to improve HIV-TB integrated service delivery.
- The QI intervention implemented dramatically improved isoniazid preventive therapy initiation rates among eligible HIV patients and resulted in moderate improvements in HIV testing and screening, TB screening, and viral load monitoring.

**Key Implications:**

- Program managers should ensure that all categories of health care workers from all levels of the health care system are included in QI workshops or learning sessions to harness the knowledge and experiences of all role players working within the system.
- QI implementers should consider adopting a combination approach to improvement interventions, such as QI training combined with mentorship, collaborative learning, and data QI activities.
- To strengthen and ensure the success of QI interventions, senior-level program managers should consider allocating resources (human, financial, and infrastructure) dedicated to data QI for a sustainable and effective QI program.

## INTRODUCTION

In South Africa, TB remains a public health challenge largely driven by a high background prevalence of HIV, estimated at 12% in the general population.^1^ In 2019, an estimated 58,000 people died from TB, of whom 36,000 (62%) were coinfected with HIV.^2^ For South Africa to achieve its goal of reducing TB deaths by 95% by 2035, steps to accelerate the reduction in TB mortality are needed, specifically in HIV-TB coinfected patients.^3^

Integrating HIV and TB services (hereafter written HIV-TB services) is a key strategy in reducing TB-related deaths among people living with HIV.^4^ HIV-TB services refers to screening, diagnosis, and treatment services provided for both diseases at the same clinic, by the same clinic team, on the same visit day.^5^^,^^6^ We have previously published the key evidence-based, clinical HIV-TB integration activities that have been shown to reduce TB-related mortality among people with HIV, TB, and both HIV and TB.^7^ Specific integration services include HIV testing services (HTS) for all TB patients, TB screening for all clinic attendees, isoniazid preventive therapy (IPT) initiation for eligible HIV patients, antiretroviral therapy (ART) and cotrimoxazole for all HIV-TB coinfected patients, and retention and treatment adherence monitoring.^7^ All HIV-TB integration activities mentioned are incorporated into the South Africa National Department of Health (DOH) HIV treatment guideline document.^8^ However, suboptimal implementation of HIV-TB services in public health facilities has been observed where opportunities to screen patients for TB, test for HIV, and subsequent linkage to treatment have been missed.^3^^,^^9^^,^^10^ While patient-related factors such as stigma and fear of HIV testing may be contributing to gaps in the HIV-TB care cascade, there is mounting concern that weaknesses in health care systems at the frontline are not adequately addressed.^11^

Operationalizing and delivering high-quality HIV-TB services is complex and challenging in resource-constrained settings.^5^^,^^12^ The need for simple, low-cost, and sustainable solutions to enhance service delivery was the impetus for introducing quality improvement (QI) methods in public health settings.^13^^,^^14^ The defining principle of QI is the focus on improving underlying health systems and addressing gaps with feasible solutions.^15^ In South Africa, QI was successfully implemented to reduce mortality in mothers, neonates, and infants.^16^^,^^17^ However, little is known of the effectiveness of QI in reducing mortality in patients accessing public health facilities for HIV, TB, and HIV-TB services.^7^

The Centre for the AIDS Programme of Research in South Africa (CAPRISA), implemented a cluster-randomized trial, the scaling up TB and HIV treatment integration (SUTHI) trial, designed to test the effectiveness of a QI intervention in enhancing HIV-TB service integration to reduce mortality in HIV-TB patients.^7^ CAPRISA, in partnership with the Institute for Healthcare Improvement (IHI), designed and implemented a QI intervention to enhance HIV-TB service delivery by identifying and addressing the health system’s weaknesses at the primary health care (PHC) clinic level.^7^

A QI intervention was implemented to enhance HIV-TB service delivery by identifying and addressing the health system’s weaknesses at the primary health care clinic level.

In this article, we describe the QI intervention, our theory of change, report the impact of the intervention on HIV-TB services, identify changes that were associated with improved processes outcomes, and elucidate challenges associated with implementing QI to improve HIV-TB services in PHC clinics.

## METHODS

### The SUTHI Trial

The design and rationale for the SUTHI trial are published elsewhere.^7^ Briefly, SUTHI was a cluster-randomized trial in which 16 PHC nurse supervisors (clusters) and the 40 PHC clinics under their oversight were randomly assigned to receive either a structured program of QI training and mentorship to expand the skill and capacity of health care workers in improving HIV-TB services (QI intervention group) or to the standard of care supervision and support (SOC) group as carried out by the South Africa DOH. Eight nurse supervisors and their 20 clinics were assigned to the QI intervention group and 8 nurse supervisors and their 20 clinics were assigned to the SOC group. All clinics were followed up for 18 months.

### Setting

The SUTHI trial was located in the King Cetshwayo District and Ugu District in KwaZulu-Natal Province, South Africa. The King Cetshwayo District and Ugu Districts have reported incident TB rates of 859 per 100,000 and 810 per 100,000, respectively; antenatal HIV prevalence rates of 33.4% and 41.7%, respectively; and mortality rates attributable to TB and HIV of 36% and 35%, respectively.^18^ Given the high rates of TB and HIV, both districts were ideal locations for the SUTHI trial. In South Africa, PHC clinics are the first point of entry into the health care system for a large majority of the population and services are free.^7^ The South African DOH HIV treatment guidelines recommends provision of integrated HIV-TB health care as standard practice.^8^

### Standard Support and Supervision

The QI intervention in the SUTHI trial was implemented in parallel to other improvement activities undertaken by the district management team (DMT). Improvement initiatives undertaken by the DMTs were considered as a SOC and were available to the QI group clinics and the SOC group clinics. Both study districts were supported by a highly motivated DMT who conducted routine, in-person, quarterly PHC clinic visits, and weekly data-driven progress update meetings with representatives from all facilities, including SUTHI study clinics. DMT involvement was maintained throughout the study period. Support from local, nongovernmental organizations (NGOs) for the improvement of the HIV and TB programs in both districts were present both before and during the study.

## CHANGE THEORY

**FIGURE 1:**
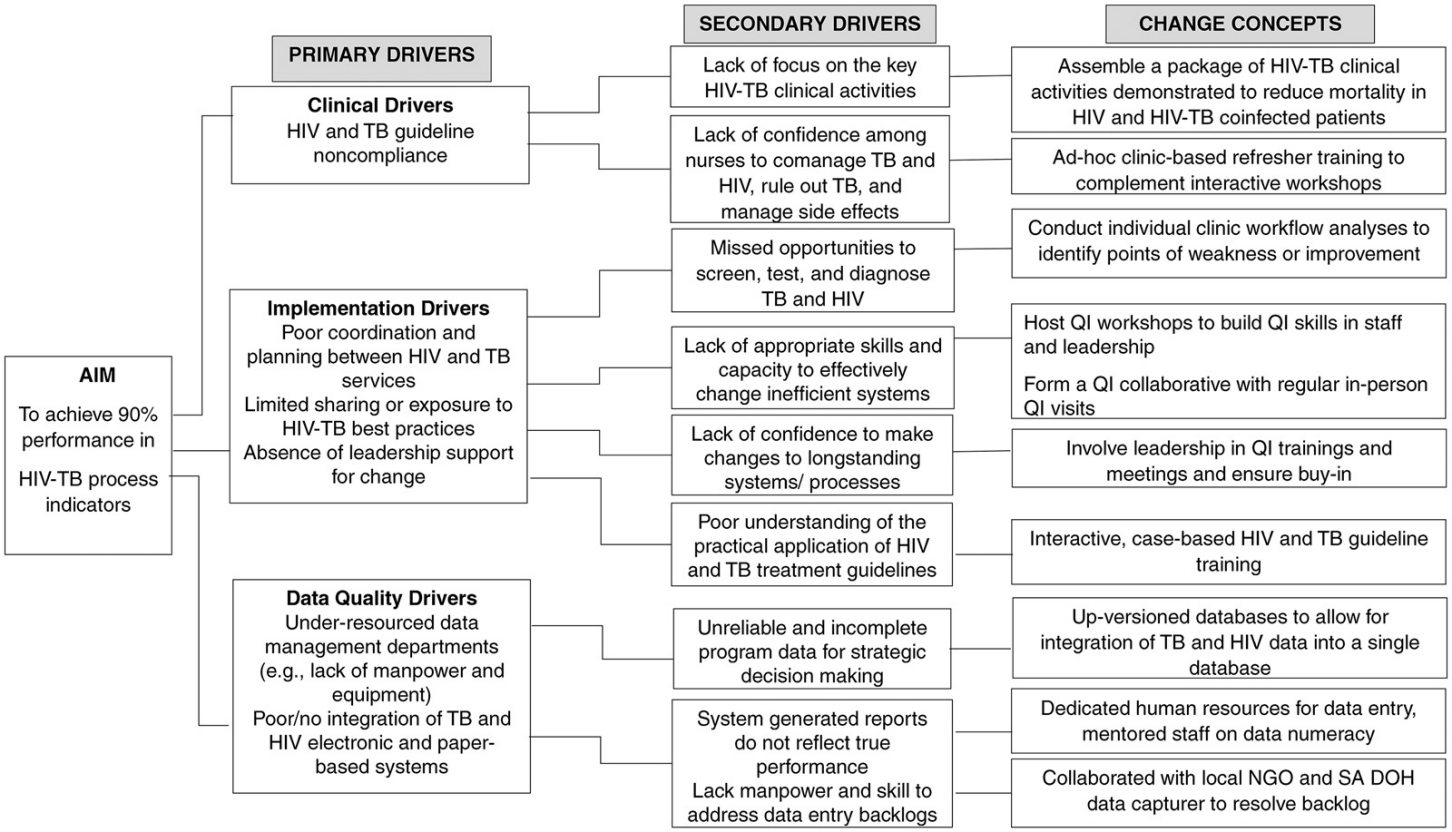
Change Theory Based on Primary and Secondary Drivers of Poor Performance in Integrated HIV-TB Services and Change Concepts Used in a Quality Improvement Intervention for HIV/TB Service Integration in KwaZulu-Natal, South Africa Abbreviations: NGO, nongovernmental organization; QI, quality improvement; SA DOH, South African Department of Health.

A change package to guide implementation of HIV-TB services was not available. Instead, we implemented an intervention that would allow change ideas to emerge from the input and experiences of the clinic staff and nurse supervisors in the QI intervention group. Our change theory was premised on a collective understanding from published articles and feedback from implementers on the primary and secondary drivers of poor performance in HIV-TB service integration (Figure 1). Primary elements of our change theory were: (1) HIV-TB clinical content comprising a package of essential evidence-based interventions supported by an implementation algorithm suitable for a clinic setting, (2) implementation content comprising health care worker training and clinical skills capacity building for improved identification and treatment of HIV-TB patients as well as training in QI methodology; and (3) data quality improvement to enhance reliability and completeness of routine HIV-TB data.

### Clinical Content

**FIGURE 2:**
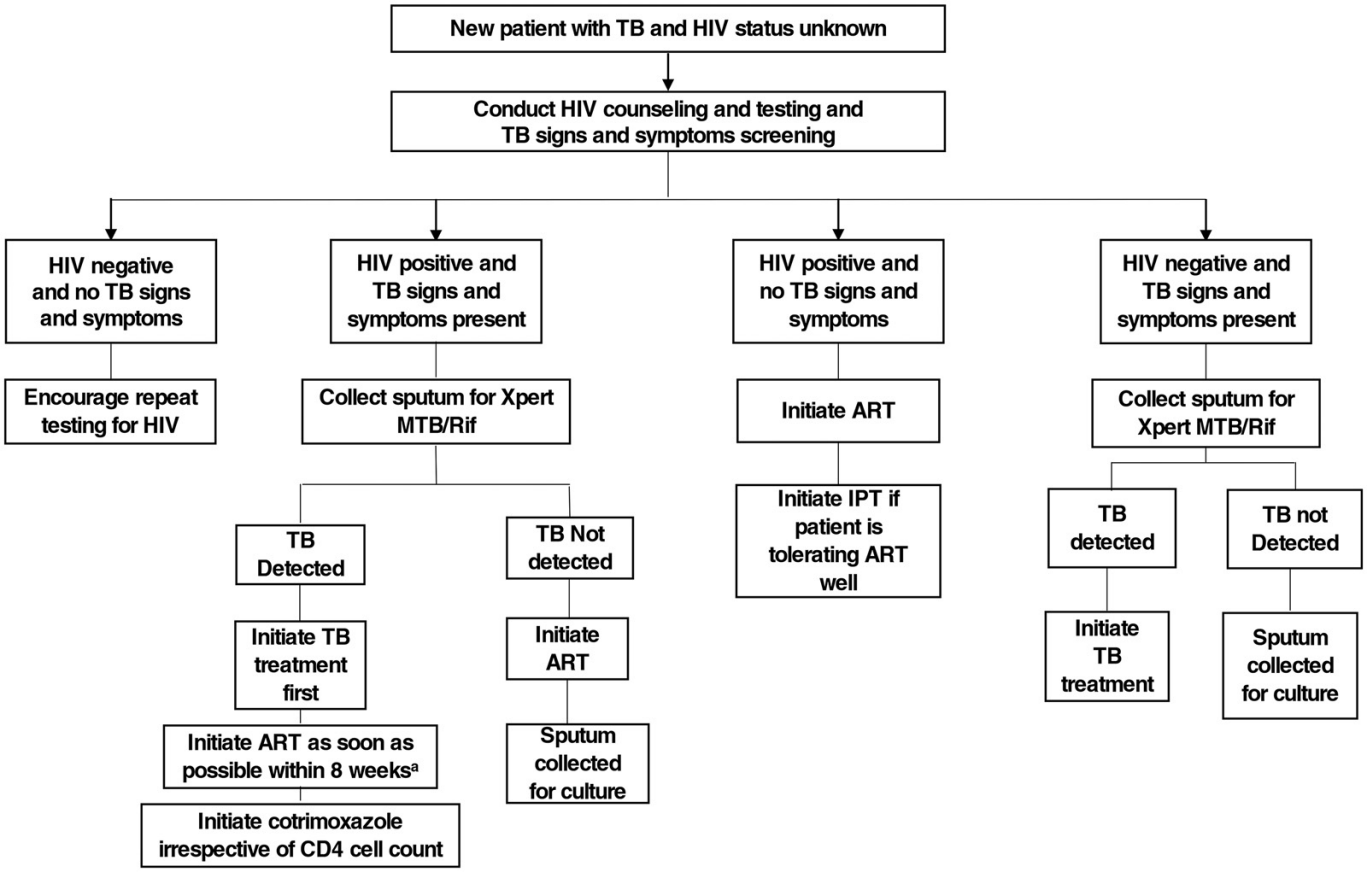
Key HIV-TB Services Care Algorithm Training Tool Used in a Quality Improvement Intervention for HIV/TB Service Integration in KwaZulu-Natal, South Africa Abbreviations: ART, antiretroviral therapy; IPT, isoniazid preventive therapy; Xpert/MTB/Rif, a rapid, molecular, cartridge-based test used for TB diagnostics that provides an immediate rifampicin resistance result. ^a^ For HIV-TB coinfected patients: If CD4<50 cells/µl, initiate ART within 2 weeks of starting TB treatment AND if CD4>50 cells/µl, initiate ART within 2–8 weeks of starting TB treatment.

The development of the package of HIV-TB services was preceded by a review of published literature, South African HIV and TB treatment guidelines and policies, and input from experts in the field of HIV-TB co-management to identify the most effective evidence-based clinical activities associated with a reduction in mortality in HIV-TB coinfected patients. We assembled key HIV-TB clinical services into an HIV-TB care algorithm (Figure 2) that served as a training tool for QI group clinics. Health care workers in the QI collaborative were trained to appropriately identify, triage, and treat HIV-TB patients and prevent TB in HIV patients.

### Implementation Content

Historically, HIV and TB services operated separately; however, the directives, policies, and guidelines from South Africa National DOH to co-locate and integrate both services at a single facility, without adequate implementation guidance, failed to integrate HIV-TB health care delivery.^5^ Efficient integration of services requires joint planning and coordination between different departments within a clinic together with the provision of relevant training.^19^ We undertook to ensure that staff had the clinical skills to find and treat HIV-TB coinfection and quality improvement skills to strengthen and optimize HIV-TB patient flow and workflow processes.

#### Improving Clinical Skills in Screening, Diagnosis, and Management of HIV-TB Coinfection

At the start of the study, a 1-day training workshop in each district was conducted for the QI collaborative with a study-appointed clinician trainer and members of the DMT serving as facilitators. The training session emphasized that integrated HIV-TB services meant delivering both HIV and TB care and treatment at the same facility, by the same clinic team on the same day, also known as “the single facility approach.”^5^ Training content included a review of the Xpert MTB/RIF algorithm for the screening and diagnosis of TB; timing and criteria for ART initiation in TB patients; HIV-TB comanagement in adults, pregnant women, and pediatrics; and utilization of data reports from routine electronic databases to track health systems performance. An interactive, case-based mode of teaching was adopted where treatment and patient scenarios resembled typical real-world situations to which the audience could relate.

#### Use of QI Methods to Improve Integrated HIV-TB Services

In this study, we used the Model for Improvement as the methodological framework to identify systems’ weaknesses and optimize workflow to enhance the performance of HIV-TB services to acceptable standards stipulated in the UNAIDS 90-90-90 strategy document (Supplement Figure 1).^20^

Each clinic in the QI collaborative reviewed their clinic patient flow to understand the system and identify weaknesses, bottlenecks, or potential improvements that would strengthen HIV-TB care delivery. The clinic QI team consisted of 1 representative from each staff category to ensure all perspectives were represented. Whenever possible, PHC clinic supervisors and clinic operations managers participated in biweekly QI meetings.

The plan-do-study-act (PDSA) cycle was the guiding framework used to test and accumulate knowledge on proposed change ideas. During the plan phase, appropriate clinic team members, who would test the change idea, were identified and roles and responsibilities explained. Change ideas were recorded and as a QI team member tested changes (do phase), other team members collected process data and made observations of any unintended or negative impacts on the system. During the study phase, annotated run charts were used to track the performance of HIV-TB service outcomes and reviewed every 2 weeks by QI clinic teams. In the act phase, the QI team decided on adapting, adopting, or abandoning change ideas. On average, 4 PDSA cycles per HIV-TB indicator were completed before a change idea was perfected and adopted.

#### Participation in a Learning Network

All nurse supervisors and clinics in the QI intervention group formed a learning collaborative that was based on an approach designed by the IHI called a Breakthrough Series Collaborative.^21^ The Breakthrough Series Collaborative operates on the principle that, when brought together, organizations working toward a common goal can accelerate learning by sharing knowledge, data, challenges, and experiences.^21^ In this study, the learning collaborative was brought together for 3 learning sessions timed at 6-month intervals from the month of study enrollment (details of the learning session content are available in Supplement Figure 2). Key elements of the learning sessions were: (1) didactic teaching emphasizing the global and local seriousness of the HIV-TB co-epidemic and the evidence for integrating HIV-TB services, (2) an analysis of local PHC clinic data and identification of gaps in meeting HIV-TB service delivery targets, and (3) interactive group sessions among clinic teams to discuss challenges and potential solutions. Two study-appointed QI nurse mentors conducted bimonthly face-to-face visits in the first 12 months and thereafter reduced to monthly face-to-face visits in the last 6 months of the study. Face-to-face visits included meeting with the clinic QI teams, observing the clinic teams in their daily routine, and ensuring implementation of QI plans.

### Improving Data Quality

A roving team of study-appointed data capturers conducted regular quality assurance checks on patient registers, chart notes, and electronic HIV and TB databases maintained at the clinic. Paper-based systems were checked for completeness, legibility, and accuracy. Every 6 months patient chart note data were compared to the electronic system data for a randomly selected sample of HIV, TB, and HIV-TB patients. Feedback on discrepancies, incorrect, or missing data was given to clinic teams. The roving team assisted with clearing major backlogs in data entry.

### Key Inputs for QI Intervention Implementation

The implementation of the QI intervention required the establishment of a partnership between CAPRISA and IHI, appropriately skilled staff to drive the QI activities, and technically skilled data staff to improve data quality.

Local QI expertise, with formal QI training and practical experience, was a scarce resource at the start of the trial. By partnering with IHI, the SUTHI trial gained an experienced leader in QI implementation methods. At the design phase of the study, IHI played a key role in training study staff in QI methods using a train the trainer model. Two study-appointed professional nurses (1 per study district) trained by IHI, drove the QI process at the clinic level and were under the oversight of a QI advisor from IHI who provided mainly virtual support. Each nurse supported 10 QI clinics. Between study enrollment to month 12 made fortnightly, the nurse made in-person mentorship visits to QI clinics. These visits were reduced to monthly mentorship visits between month 13 and month 18.

By partnering with IHI, the SUTHI trial gained an experienced leader in QI implementation methods.

A data manager based at the CAPRISA headquarters oversaw the roving data quality improvement team that consisted of 2 data coordinators (1 per district), and 6 data capturers (3 per district). The intervention was implemented in the context of a cluster-randomized trial and to ensure that we had comparable data in the QI clinics and SOC clinics, the data team conducted improvement activities in both study groups during the study. The data team made fortnightly visits to QI improvement clinics and similarly to SOC clinics.

In addition, due to the nature of the trial design, learning sessions were held in conference venues and not on South Africa DOH premises. All costs of the venues, accommodation for trainers, and transport of health care workers were borne by the study.

### Study Outcomes and Data Collection

HIV-TB process indicators were collected every month from paper-based registers (ART, TB, and HIV registers), electronic databases, and patient chart notes. These data were recorded onto paper-based data collection tools and faxed to the central office. Training registers were completed at each QI workshop, recording the number and designation of health care workers that attended. The QI nurse mentor and clinic QI team maintained detailed records on a PDSA template (provided by IHI) of the dates that QI work began per indicator and the change ideas, adaptations, and challenges encountered. The completed PDSA templates were submitted for analysis. Table 1 defines the HIV-TB process indicators that clinic teams selected for improvement and data elements used to calculate performance. For ease of reference, a shortened name (abbreviation) was assigned to each indicator in Table 1 and will hereafter be used in all subsequent sections.

**TABLE 1.** Definitions of HIV-TB Process Indicators Used in the Quality Improvement Intervention to Integrate HIV-TB Services in KwaZulu-Natal, South Africa

**HIV-TB Process Indicator**	**Abbreviation**	**Definition**
HTS for PHC clinic attendees	HTS	Percentage of patients that accessed HIV tests, expressed as a percentage of the clinics’ HIV testing target^a^ Numerator: Number of patients tested for HIV Denominator: Clinic assigned target for HTS
TB screening among PHC clinic attendees	TB screening	Percentage of clinic attendees screened for TB signs or symptoms^b^ Numerator: Number of clinic attendees screened for TB signs and symptoms (adults and children) Denominator: Clinic headcount (Number of people accessing any health services at a facility during a specified period)
Initiating IPT among eligible new ART patients	IPT initiation	Percentage of new ART patients initiated onto IPT Numerator: Number of new ART patients initiated on IPT Denominator: Number of new ART patients with no signs or symptoms of TB
ART initiation among TB/HIV coinfected patients	ART initiation	Percentage of TB/HIV coinfected patients initiated on ART Numerator: Number of TB/HIV coinfected patients initiated on ART Denominator: Number of confirmed TB patients tested positive for HIV
VL testing at month 12 after ART initiation^c^	VL testing	Percentage of eligible ART patients who had a VL test at month 12 after ART initiation Numerator: Number of ART patients who received a VL test at month 12 after ART initiation Denominator: Number of ART patients eligible for a VL test at month 12 after ART initiation

Abbreviations: ART, antiretroviral therapy; HTS, HIV testing services; IPT, isoniazid preventive therapy; PHC, primary health care; VL, viral load.

a All clinics receive a monthly target for HIV Testing Services from their respective District Offices.

b TB signs and symptom screening refers to the verbal screening checklist which documents the common signs and symptoms of TB (current cough of any duration, fever for >2 weeks, drenching night sweats, Unexplained weight loss of >1.5kg in a month).

c According to the South African National Department of Health National Consolidated guidelines, a viral load test is required at month 6 and month 12 after ART initiation and annually thereafter. This study focused on the month 12 viral load only.

### Statistical Analysis

**FIGURE 3:**
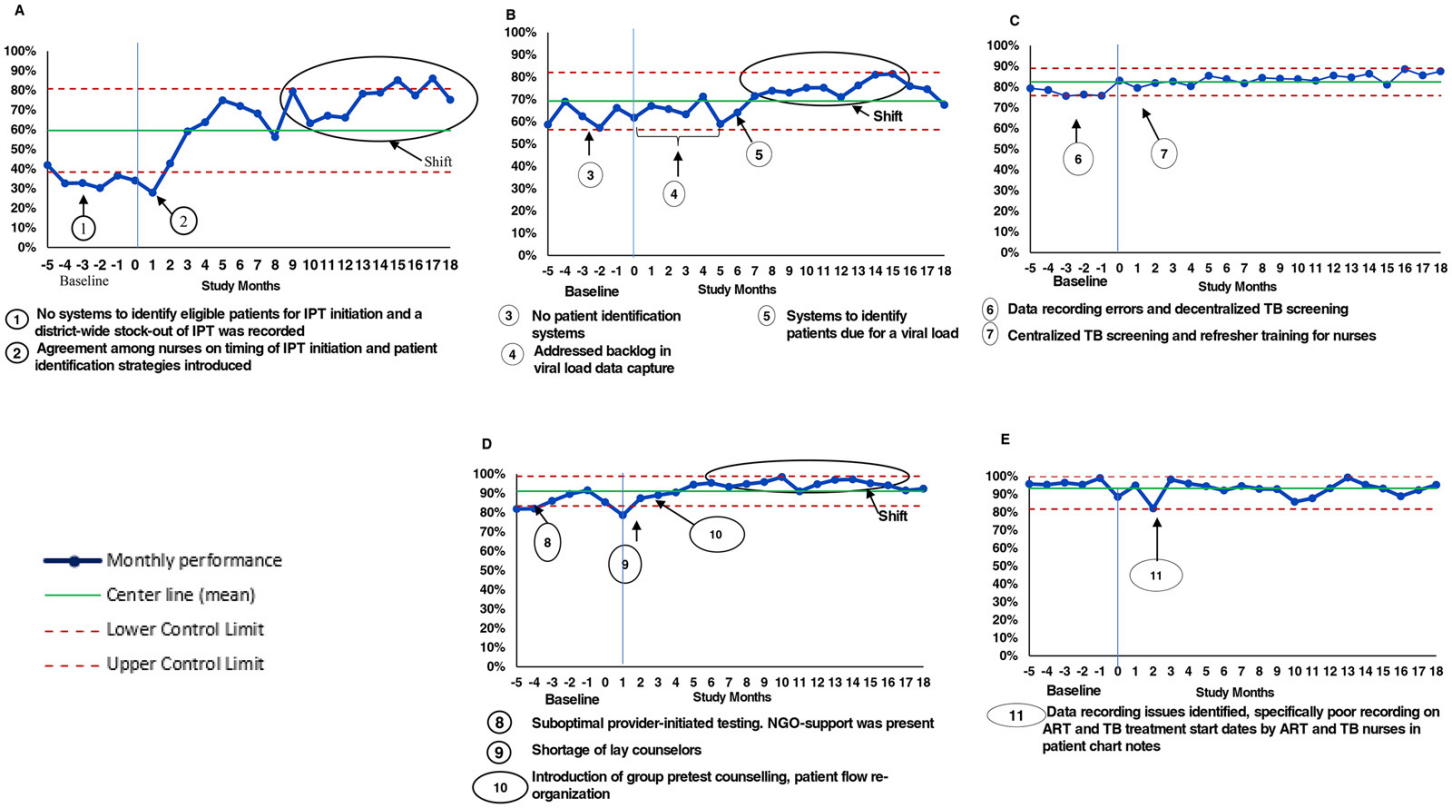
xmr Charts of Monthly Performance in HIV-TB Process Indicators in a Quality Improvement Intervention for HIV/TB Service Integration in KwaZulu-Natal, South Africa (a) Percentage of Eligible New ART Patients Initiated on IPT; (b) Percentage of ART Patients With a Viral Load Test Conducted; (c) Percentage of PHC Clinic Attendees Screened for TB; (d) Percentage of HIV Target Achieved; (e) Percentage of HIV-TB Coinfected Patients Initiated on ART Abbreviations: ART, antiretroviral therapy; IPT, isoniazid preventive therapy; PHC, primary health care.

We analyzed data at the nurse supervisor level (the cluster). Monthly performance for each HIV-TB process outcome was calculated by summation of numerators of all clinics that comprised a cluster and divided by the sum of the denominators of all respective clinics in the cluster. The mean of all cluster means reflected the monthly performance, which was then plotted as *xmr*-charts (Figure 3). A run of 8 or more data points on 1 side of the center line was defined as a shift and a run of 8 or more data points in an upward or downward direction was defined as a trend.^22^ Geometric means were calculated as a single estimate of baseline performance (last 6 months before study enrollment) and for the post-QI intervention phase (months 13–18) (Table 2). The absolute difference between the post-QI intervention geometric mean and the baseline geometric mean was calculated to reflect the size and direction of the improvement. Paired *t*-test was used to determine if differences between baseline and post QI intervention phases were statistically significant for each indicator. Completed PDSA templates were examined by 2 study staff members and common systems weaknesses and associated change-ideas were identified and summarized.

**TABLE 2.** Summary of Changes in HIV-TB Process Indicators Used in the Quality Improvement Intervention to Integrate HIV-TB Services in KwaZulu-Natal, South Africa

**HIV-TB Process Outcomes**	**Proportions (95% CI)**		**Absolute** **Difference**	***P* Value**	**Clinics**^a^ **(N=20)**	**PDSA Cycles** **Mean, (Range)**
	**Baseline**	**Post-QI Intervention**				
HTS	84.8 (75.5,95.3)	94.5 (89.3,99.9)	9.7	.110	12	3 (1–7)
TB screening	76.2 (65.4, 88.9)	85.2 (78.7,92.2)	9.0	.040^b^	17	4 (1–9)
IPT initiation in new ART patients	15.9 (4.8,52.5)	76.4 (66.3,88.1)	60.5	.019^b^	20	4 (1–11)
ART initiation in HIV-TB patients	95.8 (93.3,98.3)	94.1 (89.7,98.6)	−1.7	.481	3	1 (1–3)
Viral load monitoring	61.4 (56.4,66.8)	74.0 (65.5,83.6)	12.6	.045^b^	20	4 (1–7)

Abbreviations: ART, antiretroviral therapy; CI, confidence interval; HTS, HIV testing services; IPT, isoniazid preventive therapy; PDSA, plan-do-study-act; QI, quality improvement.

a Number of clinics engaged in quality improvement.

b *P* value significant at <.05 using paired *t*-tests.

### Ethics

The SUTHI trial was approved by the Biomedical Research Ethics Committee of the University of KwaZulu-Natal (BREF Ref 108/14). Informed consent for the study was waived.

The KwaZulu-Natal Health Research and Knowledge Management committee granted permission to access PHC clinics in the study districts of KwaZulu-Natal (HRKM309/14).

## RESULTS

The QI intervention was conducted from December 1, 2016, to January 1, 2019. Table 3 provides a summary of health care workers who attended the 3 learning sessions. At no learning session were all 8 PHC clinic supervisors present.

**TABLE 3.** District and Clinic Staff Trained in Quality Improvement Methods for a Quality Improvement Intervention for HIV/TB Service Integration in KwaZulu-Natal, South Africa

**Staff Category**	**Pool of Health Care Workers Available** **N=259**	**Actual Number Trained in QI**		
		**Learning Session 1** **N = 63**	**Learning Session 2** **N=61**	**Learning Session 3** **N = 45**
		**n (%)**	**n (%)**	**n (%)**
**District Management Team**				
TB program manager	3	2 (3)	1 (2)	2 (4)
HIV/AIDS/Sexually transmitted infection and TB manager	2	2 (3)	2 (3)	2 (4)
Training coordinator	2	2 (3)	2 (3)	0
Nurse supervisors	8	5 (8)	4 (6)	3 (7)
**Subtotal**	**15**			
**Clinic Staff Categories**				
Operations managers	19	11 (17)	9 (15)	9 (20)
Professional nurses	85	6 (10)	11(18)	8 (18)
Enrolled nurses/ enrolled nurse assistants	61	8 (11)	6 (10)	1(2)
Data capturers	36	17 (27)	18 (30)	19 (42)
Lay counselors	43	10 (16)	8 (13)	1 (2)
**Subtotal**	**244**			

Clinic QI teams identified HIV-TB processes for improvement based on findings of patient- and work-flow analyses and suboptimal performance at baseline (Table 4). Systems weaknesses and opportunities for improvement were identified in all clinics for IPT initiation and VL testing at month 12 after ART initiation. However, HTS, TB screening, and ART initiation became the foci of QI initiatives in, 17 and 3 clinics, respectively. Clinics that did not actively engage in improving an indicator continued to monitor performance only. All clinics were included in analyses of the performance of the collaborative.

**TABLE 4:** Health Systems Weaknesses Identified and Associated Change Ideas for a Quality Improvement Intervention for HIV/TB Service Integration in KwaZulu-Natal, South Africa

**HIV-TB Process**	**Health Systems’ Weaknesses Identified**	**Change Concepts**
**HTS**	Relying only on patient requests or referrals for HIV testing.	**Introduced strategies to enhance provider-initiated testing:** Offered group pretest counseling in all patients’ waiting areas Implemented a daily roster system of staff to conduct pre-test counseling Nurse in charge or designee to check accountability log daily
	Missed opportunities to offer HTS to all patients Acute patients were overlooked for HTS services (e.g., wound care patients)	**Redesigned clinic patient flow** Ensure that acute patients are directed to lay counselors after vitals assessments^a^ are conducted If above not possible, then staff caring for acute patients were (i) trained in HIV testing and counseling and (ii) provided with the appropriate HTS stationery
	HTS data inaccuracies caused by: Not completing HTS registers in real-time Misplacing HTS registers	**Daily data quality control checks** Daily quality control of HTS registers and frequent audits of patient files and electronic data to ensure HIV status is known for all patients
	Overdependence on lay counselors HTS viewed as the work of lay counselors Lack of counseling skills among nurses to relieve/stand-in for lay counselors	**Increasing the accountability and responsibility for the HTS program** On-site HTS refresher training was held which addressed: pre- and post-test counseling messages, conducting HIV rapid tests, and data recording Awareness of clinic target set by the district health office was disseminated
**TB screening among PHC clinic attendees**	Missed opportunities to offer TB screening to all clinic attendees	**Centralized TB screening** Made TB screening mandatory at an identified strategic point visited by all patients, such as, vitals assessment^a^ station Visual prompts and reminders to conduct TB screening included large and colorful TB posters, printed and easily accessible signs, and symptoms checklists Made TB screening mandatory for acute patients
	Inaccurate TB screening data	**Data quality control checks** Daily data quality control checks conducted by nurse in charge or designee to check: Completeness and accuracy of daily TB screening register Number of symptomatic patients and number of sputum samples sent for Xpert/ MTB Rif^b^ testing Quality control of clinic headcount^c^ data: Exclude patient representatives or family members Subtract TB confirmed patients from the clinic headcount Mass TB screening campaigns conducted in communities must be distinguishable from screening conducted in the clinic
**IPT initiation among new ART patients**	Ambiguity in IPT initiation guidelines Nurses lack clarity on timing of IPT initiation Individual nurses use own discretion to start IPT	**Clarify IPT initiation timing and arrive at mutually agreed upon timing for initiation** Each clinic team arrived at a common time to start IPT (e.g., 7,14, or 30 days after starting ART) Agreed upon timing was documented and standardized for entire clinic
	Confusion about roles and responsibilities of clinic staff	**Enhancing accountability and responsibility for IPT program** Roles and responsibilities were assigned to all staff categories and documented
	No system for identifying patients eligible for IPT	**Strategies to identify patients returning at the agreed upon time for IPT** The “box system” -eligible patients’ files placed in a decorated box for easy identification, OR Tagged files of eligible patients with stickers or red ink OR The “diary system” reminder note in clinic diary to initiate IPT at next visit and note attached to patient file
	Poor recording of IPT initiation date in clinic chart notes	**Refresher training on clinic stationery to document IPT** Nurses directed to document start date in designated fields and data capturers shown where to find the start date
	Nurses lack confidence to rule out TB	**Host a training for nurses, lay counselors, and data capturers highlighting the importance and potential benefit of IPT for HIV-infected patients** Link this training with the TB screening training (above) to boost confidence to rule out TB
**ART initiation among HIV-TB coinfected patients**	**Patient chart notes for TB and ART kept separately** TB and ART files not integrated No unique identifier for TB and ART file	**Combining ART and TB files** For HIV-TB coinfected patients, ART and TB chart notes were physically combined The district health office agreed upon a common unique identifier to be used The TB module on the electronic ART database was activated to accommodate TB and ART data
	Poor coordination between NIMART and TB nurses regarding ART and TB treatment initiation	**Refresher training for nurses** Clarified patient flow for ART initiation visits in TB/HIV coinfected patients Improved chart notes for ART and TB treatment start dates
**VL monitoring at month 12 after ART initiation**	No system to identify patients eligible for month 12 VL tests	**Generate report from electronic system of patients due for VL** Address the data capturing backlog of VL results and ART initiation Draw on the assistance of local nongovernmental organizations and support partners for assistance with data capture Generate VL reports from the data system to determine which patients have not had or are due for VL test (filter out deceased and transferred-out patients) Tag/mark the files of patients due for VL for easy identification Trace patients who were missed for a VL test

Abbreviations: ART, antiretroviral therapy; HTS, HIV testing services; IPT, isoniazid preventive therapy; NIMART, Nurse Initiated Management of Antiretroviral Therapy; PHC, primary health care; VL, viral load.

a Vitals assessments refers to general measures of well-being which typically include weight, body temperature, blood pressure measurements.

b Xpert/MTB RIF a rapid, molecular, cartridge-based test used for TB diagnostics that provides an immediate rifampicin resistance result.

c Clinic headcount refers to the total number of patients who accessed the clinic for any type of clinical service.

IPT initiation at baseline was 15.9% (95% confidence interval (CI)=4.8,52.5) (Table 2). The main causes of poor IPT initiation were identified as uncertainty among nurses on timing of IPT initiation in new ART patients and weak systems to identify returning ART patients who were eligible for IPT (Table 4). The improvement in IPT initiation observed after the start of the QI intervention was due to a district-level IPT stock-out in the baseline period. (Figure 3A). By study month 6, a 64.8% IPT initiation rate was achieved. In the last 6 months of the study, the QI collaborative achieved a mean of 76.4% (95% CI=66.3,88.1), a 5-fold higher mean than at the baseline phase, *P*=.019 (Table 2). On average clinics carried out 4 PDSA cycles to improve IPT initiation, and while major improvement was observed, the target of 90% was never attained in the study. Improvement in IPT performance is observed from month 1; however, a shift above the mean was observed from month 9 to 18 (Figure 3A).

At baseline, the mean rate of VL testing was 61.4% (95% CI=56.4,66.8), 28.6% below the 90% desired target. Major backlogs in VL data entry that generated inaccurate VL completion reports were the main cause of poor performance identified by the QI teams. In the first 6 months post-study enrollment, no QI activities were recorded in any QI clinics to improve VL, instead, efforts to reduce the data entry backlog for the last 12 months were undertaken and QI activities were started closer to study month 6 (Figure 3B). A shift above the mean was observed from month 6 to 16. During the last 6 months of the study, the mean VL monitoring rate was 12.6% higher than the baseline rate (*P*=.045), which was less than 50% of what was needed to meet the target (Table 2).

Data inaccuracies were noted at baseline for TB screening (Figure 3C). Data quality checks and refresher training were change ideas tested for improvement (Table 4). Mean TB screening rates improved by 9% between the baseline period and post-QI intervention (Table 2), and the 90% target was not achieved by the collaborative.

Mean ART initiation rates were greater than 90% at baseline and continued post-intervention period (Table 2). The monthly performance in ART initiation was addressed only in 3 clinics, and the decrease of 1.7% was not significantly different from baseline performance (*P*=.481). HTS was the only indicator that was improved and exceeded the 90% target (Figure 3D, Table 2).

## DISCUSSION

This article describes the QI intervention implemented in the SUTHI cluster-randomized trial to improve HIV-TB health care performance. In South Africa, integrated HIV-TB services are mandatory, and this study shows that improvement in HIV-TB process outcomes is needed and possible. Using the Model for Improvement, we showed that IPT initiation improved substantially; whereas HIV testing, TB screening, and VL monitoring were moderately improved, and ART initiation among HIV-TB coinfected patients was an already well-performing indicator that required monitoring and only a few clinics had to strengthen coordination between the TB nurses and ART-initiating nurses. An important output of the QI intervention was a set of change ideas that are potentially transferrable to other settings and could contribute to the improvement of integrated HIV-TB services.

An important output of the QI intervention was a set of change ideas that are potentially transferrable to other settings and could contribute to the improvement of integrated HIV-TB services.

Several factors can be attributed to the success of IPT initiation rates in this study. First, clarifying nonspecific initiation guidelines improved decisiveness among nurses in the timing of IPT initiation. Second, as IPT is an indicator monitored at the district and provincial levels, clinic staff were motivated to improve IPT performance. Third, low performance at baseline (15.9%), increased the likelihood and potential for improvement. Fourth, improving IPT initiation and data completeness in patient files and on IPT dispensing and stock charts, subsequently improved the IPT supply chain. The supply of IPT depends on demand for IPT. Improved IPT dispensing data provided a better reflection of the clinics' demand for IPT, and the ordering of stock was adjusted accordingly. Interestingly, approximately 6 months of QI to improve IPT and HTS was undertaken before the shift was observed. This may indicate that clinics require approximately 6 months to completely embed new processes into the clinic.

Three systematic reviews evaluating the effectiveness of QI collaboratives concurred that the size of improvement observed is often a function of baseline performance and low-performing indicators are more likely to have larger improvement.^23^^,^^24^ A QI approach to improving IPT initiation was successful in other resource-constrained settings. In a Namibian case study of QI capacity development, IPT initiation resulted in a 12% increase (from 16% to 28%) at a national level.^25^ In a Nigerian case study, situated at a single state-run hospital, IPT initiation improved by 39% (11% to 50%).^26^ Interestingly, the Namibian study was at a national level and the Nigerian study was conducted at 1 facility.^25^^,^^26^ Similar to the SUTHI study, the Nigerian study was more active in addressing issues of organization, process, management, and systems. The authors surmise that root cause analysis and first-hand involvement of clinic staff in developing systems played a role in achieving improvement.^26^

A systematic review of strategies to improve health care performance showed that large improvements (defined as 20–30 percentage point improvement) are generally achieved in strategies that used a combination of training, collaborative learning, supervision, and improvement of infrastructure (such as data quality improvement), as was done in the SUTHI trial.^27^ Yet, provider-initiated HIV testing and TB screening achieved modest improvement (defined as 5–10 percentage points). VL monitoring moderately improved from baseline (defined as between 10–20 percentage points) and ART initiation slightly decreased. These results are evidence that other factors drive the success of an improvement strategy. The role of contextual factors in influencing improvement outcomes is emerging as an important consideration when assessing QI initiatives.^28^^,^^29^ Work culture, access to knowledge resources, QI leadership, supportiveness of work environments, and staff motivation and willingness to question the status quo, are but a few examples of contextual factors that may influence the success of QI initiatives.^28^^,^^30^^,^^31^

The role of contextual factors in influencing improvement outcomes is emerging as an important consideration when assessing QI initiatives.

### Lessons Learned

In the SUTHI trial, we identified important factors that may explain the suboptimal improvement for some indicators. The effect of baseline performance was to the advantage of IPT improvement; however, HIV testing services and ART initiation in HIV-TB coinfected patients were high at baseline, and there was little room for improvement thereafter. Future QI interventions should consider baseline performance when setting expectations for improvement, however, we do not recommend that baseline performance be considered the sole criteria for selecting indicators for improvement. This study showed that there are indicators that are close to reaching targets but appear to be plateauing, for example, VL testing (Figure 3B) and TB screening (Figure 3C). QI improved both indicators and still has a role to play in addressing the barriers that prevent these indicators from reaching the desired target of 90%.

Capacitating clinic staff with data analytic skills is an important factor in ensuring the success of QI interventions because it improves technical skills, confidence, and self-efficacy of clinic teams.^32^ In addition, monitoring improvement using routine data is fundamental to the QI intervention. Learning sessions covered the basics of how routine data can be analyzed (e.g., calculation of percentages, means, and medians), plotted onto run charts, and interpreted using run-chart rules. QI mentors reinforced this knowledge at QI mentorship visits. Poor data quality threatens clinic teams’ efforts to monitor improvement and is a barrier to successful QI implementation.^33^ Despite our attempts to address the completeness and accuracy of routine data, poor data quality undermined our QI intervention. For example, TB screening data were adversely impacted by inflated clinic headcount numbers (the denominator), incorrect completion of TB screening registers, and misplaced TB registers. The success of VL monitoring improvement depended on accurate and complete data entered into the patient electronic database; however, nearly 6 months of addressing data entry backlogs reduced the time available to improve the indicator coupled with challenges of tracing of patients to return to the clinic. Tracking patients is a resource-intensive effort due to poor telephonic services, lack of vehicles, and incomplete patient contact information (namely, telephone/mobile data, lack of street addresses).

Despite our attempts to address the completeness and accuracy of routine data, poor data quality undermined our QI intervention.

A Ugandan-based QI project that aimed to improve TB case notification also relied on routine data to monitor improvement and went beyond checking clinic registers for completeness and accuracy.^34^ A data tool was used to triangulate patient data from multiple sources, that is, laboratory data, patients’ chart notes, and TB laboratory data.^34^ Unfortunately, no data metrics were available to quantify the extent to which data was improved. A South African QI project to prevent mother-to-child transmission of HIV in labor wards used a specially designed checklist that included prompts for nurses to complete and document important tasks.^17^ Following this intervention, there was a marked improvement in data quality with erroneous data, namely, percentages greater than 100% being eliminated. These studies demonstrate that innovative measures are needed to improve the quality of routine data and adding additional human resources to improve the completeness and accuracy of data may not be sufficient.

We intended for PHC clinic supervisors to lead the QI intervention, but their involvement was limited by their workloads and conflicting meetings. Clinic staff selected to attend the learning sessions did not always pass on their learnings from the workshops to their colleagues and the study-appointed nurse mentors reported resistance from non-workshop attendees to the workflow changes. A mixed-methods study identified personal- and work-environment-related factors that influence a health care worker’s ability to transfer knowledge from QI trainings to peers.^35^ Health care workers that are successful in transferring training knowledge have a positive attitude to implementing changes, interpersonal skills to address resistance from peers, and the ability to question the status quo.^35^ A work environment in which teams are receptive to new ideas, supportive of change, and leadership support is present, facilitates the transfer of training knowledge. ^35^ In the SUTHI trial, selection of clinic staff to attend learning sessions was at the discretion of the PHC clinic nurse supervisor and nurse in charge of the clinic. While individuals from all clinic departments were trained, staff categories, such as data capturers and lay counselors may not have been empowered enough to transfer their new knowledge to more senior colleagues. Future QI interventions must consider screening potential QI trainees for the appropriate qualities that will allow for the transfer of QI knowledge.

Future QI interventions must consider screening potential QI trainees for the appropriate qualities that will allow for the transfer of QI knowledge.

### Challenges in QI Implementation

Implementation of QI at the clinic level was accompanied by several challenges. First, QI was vaguely understood in both districts and clinic teams often believed that they were implementing QI by virtue of the weekly nerve center meetings and discussing problems and challenges at staff meetings. The need for the SUTHI QI intervention was initially unclear to QI clinics. The learning sessions established the importance of using a QI approach that is guided by a framework (Model for Improvement and PDSA), uses tools (e.g., process charts), locally developed strategies (change ideas), and monitoring progress with data. Importantly, the consistent visits and mentorship by the QI nurse mentors were critical in demonstrating how the frameworks and tools translated to practice.

Consistent visits and mentorship by the QI nurse mentors were critical in demonstrating how the frameworks and tools translated to practice.

Secondly, QI implementation adds additional work for clinic staff, in that data needed to be collected and recorded to track progress more frequently. While change ideas were implemented, it was a challenge to keep staff motivated to track their performance. For example, in HTS, group pretest counseling was a key change idea; however, the source documents developed to track the number of group pretest counseling sessions in patients’ waiting areas were not completed.

Third, leadership at the clinic level was supportive of the QI intervention; however, due to many commitments in and outside of the clinic, there was little involvement of clinic leaders in the QI meetings. This delayed implementation of some change ideas, as junior-level clinic staff do not have major decision-making power to make changes, such as in clinic patient flow.

### Limitations

The study had limitations. First, while the QI intervention was implemented in the context of a randomized controlled trial, we were unable to prevent exposure of QI clinics from other improvement interventions to enhance integrated HIV-TB services, particularly, improvement efforts of the DMTs and technical assistance from local NGOs. Motivated DMTs in both study districts frequently monitored the progress of HIV and TB process indicators, fed back to poorly performing clinics, and conducted quarterly in-person visits to all clinics. The true effect of the QI intervention has likely been masked by the improvement efforts of the DMTs and local NGOs. Although the study was unable to separate the effect of the DMTs’ efforts and QI intervention efforts on improvements observed, the baseline period (Figure 3A–3E) offers some insight into the performance before and after the QI intervention was implemented. The QI intervention ideally complemented the performance monitoring and feedback efforts of the DMTs which were seldom able to conduct in-depth root cause analyses of systems weaknesses and develop clinic-specific interventions. Second, the study was not adequately resourced to determine if improvements in the QI clinics were sustained beyond the study period or if the QI tools, strategies, and best practices were scaled up to more clinics in other areas. Staff turnover and changes in key personnel, who were trained in QI methods, may add to the challenge of sustaining and scaling up QI activities once the study ended. Third, as per the study design, all analyses were at the cluster level and clinics within each cluster were considered as 1 unit. However, the QI intervention was at the clinic level, and different clinics within a cluster adopted different change ideas (such as the different timing of IPT initiations in Table 4), and we could not compare clinics to determine which change ideas translated to larger improvements.

## CONCLUSION

This study showed that a QI approach to improving HIV-TB health care delivery is feasible and uptake of QI among clinic teams is evident across all indicators. With guidance, clinic staff can reveal weaknesses and gaps known only to the people who work within a system. Baseline performance of an indicator should be considered when setting expectations and assessing the size of improvement. Efforts to improve the quality of routine HIV and TB data need to be intensified for future QI efforts to be successful. The importance of basic clinical skills training should not be underestimated; however, innovative approaches to teaching health care workers need to be introduced for information to be retained and facilitate practical application. Lastly, QI complements the efforts of local NGOs and routine monitoring activities of the South Africa DOH.

## Supplementary Material

21-00157-Gengiah-Supplement.pdf

## Notes

**Peer Reviewed**
