# Supplementary material for: A Quality Improvement Intervention to Inform Scale-Up of Integrated HIV-TB Services: Lessons Learned From KwaZulu-Natal, South Africa
Source: Glob Health Sci Pract. 2021 Sep 30;9(3):444–58. doi: 10.9745/GHSP-D-21-00157 (PMC8514040; doi:10.9745/GHSP-D-21-00157)

Supplement Figure 1: Example of how elements of the Model for Improvement were addressed in the study

Model for Improvement

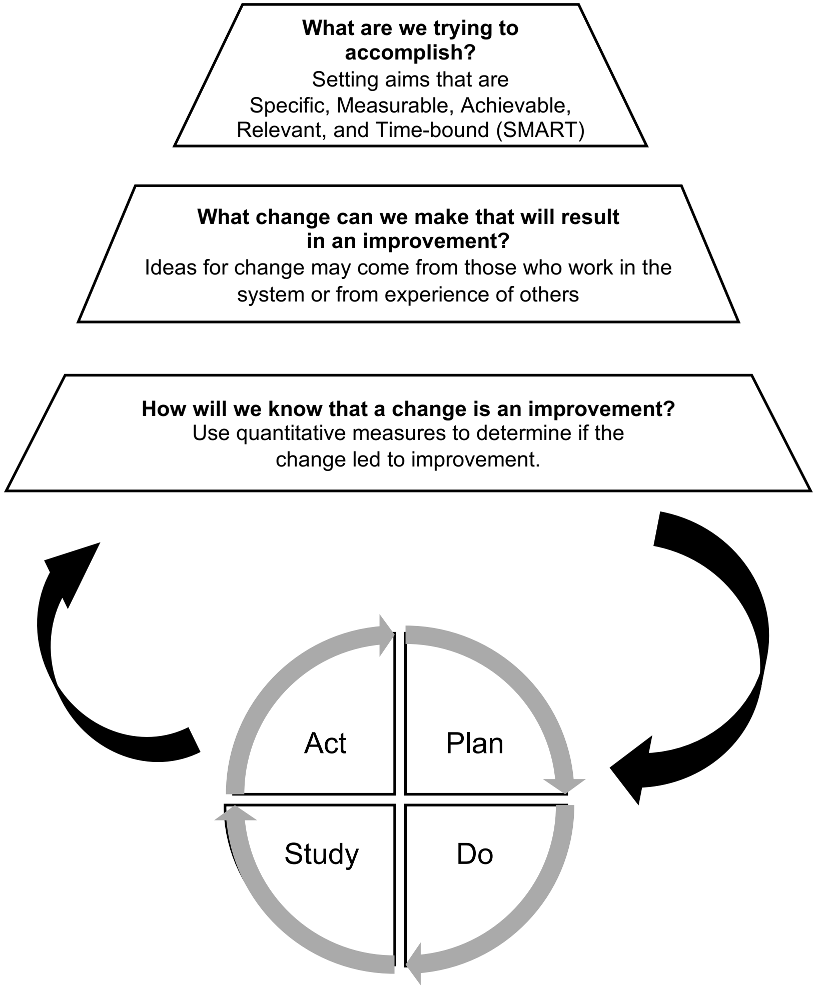

Source:
http://www.ihi.org/resources/Pages/HowtoImprove/ScienceofImprovementHowtoImprove.aspx

Example : The model for improvement implemented in a study clinic to improve Isoniazid Preventive Therapy Initiation

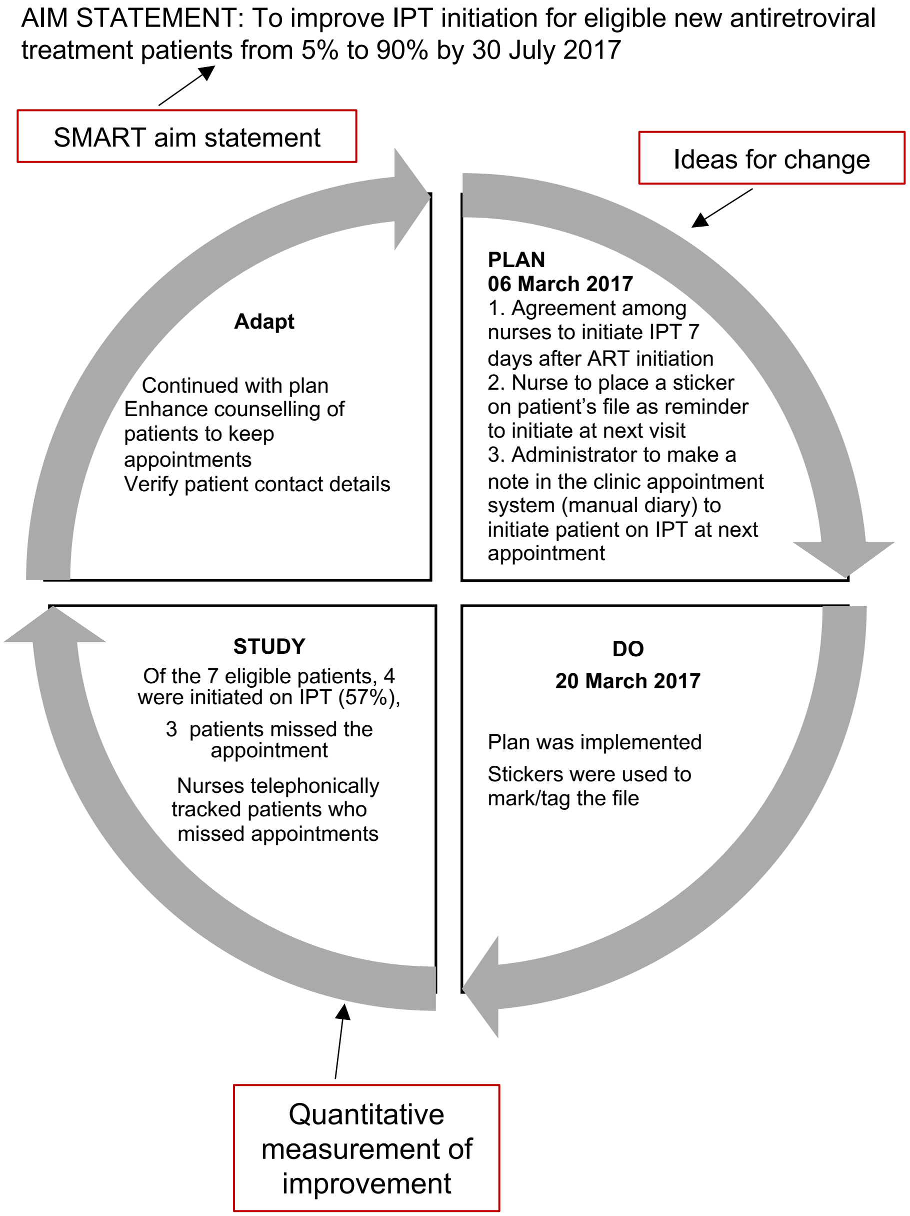

Supplement Figure 2: The Breakthrough Series approach adopted in the CAPRISA SUTHI Trial

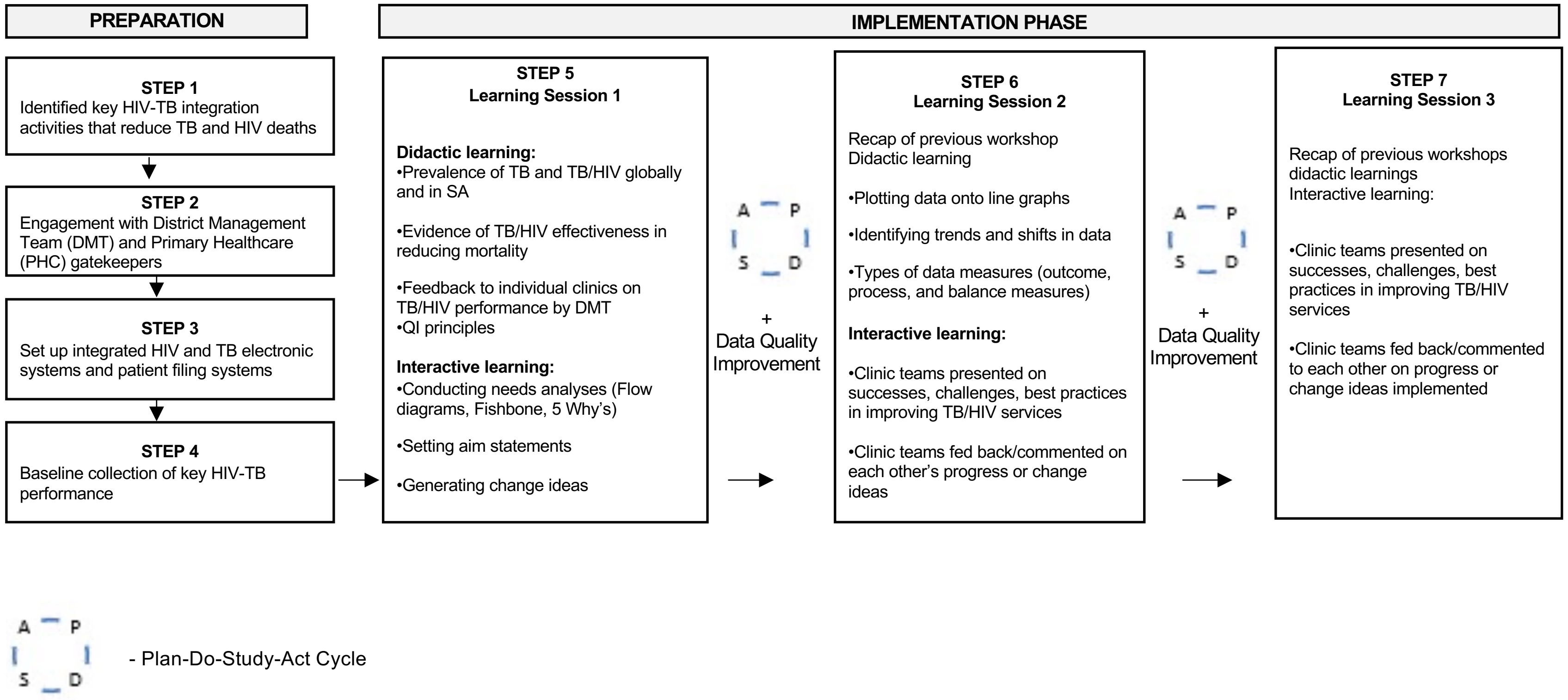

Supplement: 21-00157-Gengiah-Supplement.pdf [file 21-00157-Gengiah-Supplement.pdf]
